# Booster vaccination protection against SARS-CoV-2 infections in young adults during an Omicron BA.1-predominant period: A retrospective cohort study

**DOI:** 10.1371/journal.pmed.1004153

**Published:** 2023-01-10

**Authors:** Jiayue Wan, Casey L. Cazer, Marin E. Clarkberg, Shane G. Henderson, Scarlett E. Lee, Genevive R. Meredith, Marwan Osman, David B. Shmoys, Peter I. Frazier

**Affiliations:** 1 School of Operations Research and Information Engineering, Cornell University College of Engineering, Ithaca, New York, United States of America; 2 Department of Public and Ecosystem Health, Cornell University College of Veterinary Medicine, Ithaca, New York, United States of America; 3 Department of Population Medicine and Diagnostic Sciences, Cornell University College of Veterinary Medicine, Ithaca, New York, United States of America; 4 Institutional Research and Planning, Cornell University, Ithaca, New York, United States of America; 5 Department of Microbiology and Immunology, Cornell University College of Veterinary Medicine, Ithaca, New York, United States of America; 6 Master of Public Health Program, Cornell University, Ithaca, New York, United States of America; 7 Cornell Atkinson Center for Sustainability, Cornell University, Ithaca, New York, United States of America; University of Bern Institute of Social and Preventive Medicine: Universität Bern, SWITZERLAND

## Abstract

**Background:**

While booster vaccinations clearly reduce the risk of severe Coronavirus Disease 2019 (COVID-19) and death, the impact of boosters on Severe Acute Respiratory Syndrome Coronavirus 2 (SARS-CoV-2) infections has not been fully characterized: Doing so requires understanding their impact on asymptomatic and mildly symptomatic infections that often go unreported but nevertheless play an important role in spreading SARS-CoV-2. We sought to estimate the impact of COVID-19 booster doses on SARS-CoV-2 infections in a vaccinated population of young adults during an Omicron BA.1-predominant period.

**Methods and findings:**

We implemented a cohort study of young adults in a college environment (Cornell University’s Ithaca campus) from a period when Omicron BA.1 was the predominant SARS-CoV-2 variant on campus (December 5 to December 31, 2021). Participants included 15,800 university students who completed initial vaccination series with vaccines approved by the World Health Organization for emergency use, were enrolled in mandatory at-least-weekly surveillance polymerase chain reaction (PCR) testing, and had no positive SARS-CoV-2 PCR test within 90 days before the start of the study period. Robust multivariable Poisson regression with the main outcome of a positive SARS-CoV-2 PCR test was performed to compare those who completed their initial vaccination series and a booster dose to those without a booster dose.

A total of 1,926 unique SARS-CoV-2 infections were identified in the study population. Controlling for sex, student group membership, date of completion of initial vaccination series, initial vaccine type, and temporal effect during the study period, our analysis estimates that receiving a booster dose further reduces the rate of having a PCR-detected SARS-CoV-2 infection relative to an initial vaccination series by 56% (95% confidence interval [42%, 67%], *P* < 0.001). While most individuals had recent booster administration before or during the study period (a limitation of our study), this result is robust to the assumed delay over which a booster dose becomes effective (varied from 1 day to 14 days). The mandatory active surveillance approach used in this study, under which 86% of the person-days in the study occurred, reduces the likelihood of outcome misclassification. Key limitations of our methodology are that we did not have an a priori protocol or statistical analysis plan because the analysis was initially done for institutional research purposes, and some analysis choices were made after observing the data.

**Conclusions:**

We observed that boosters are effective, relative to completion of initial vaccination series, in further reducing the rate of SARS-CoV-2 infections in a college student population during a period when Omicron BA.1 was predominant; booster vaccinations for this age group may play an important role in reducing incidence of COVID-19.

## Abstract 2

Jiayue Wan and colleagues estimate the impact of COVID-19 booster doses on SARS-CoV-2 infection in a vaccinated population of young adults during an Omicron BA.1-predominant period.

## Author summary

**Why was this study done?:**

- In late 2021, government and health organizations were developing recommendations for Severe Acute Respiratory Syndrome Coronavirus 2 (SARS-CoV-2) booster vaccination for at-risk groups and the general public.
- University students are a generally healthy population, but high rates of SARS-CoV-2 infections occur on college campuses.
- Understanding whether booster vaccination reduces SARS-CoV-2 infections, specifically the Omicron variant, among university students will help universities and others decide whether to recommend or require booster vaccination in this population to prevent spread of disease to the local community and disruptions to the school year.

**What did the researchers do and find?:**

- We collected data on SARS-CoV-2 infections and vaccination status of over 15,000 students enrolled in mandatory testing at one university.
- We created a statistical model to compare SARS-CoV-2 infection rates among university students who had received a booster vaccination prior to an Omicron variant outbreak and students who had completed an initial vaccination series but did not receive a booster vaccination.
- The statistical model accounted for differences in infection risk due to participation in social organizations and athletic teams, sex, time during the study period, and initial vaccination date.
- Students who received a booster were approximately half as likely to get infected as students who had completed their initial vaccination series but not received a booster.

**What do these findings mean?:**

- Booster vaccination may be a useful tool to reduce SARS-CoV-2 infections on university campuses.
- Our analysis was initially performed as part of an institutional response and thus did not have an a priori protocol or statistical analysis plan, which should be considered in interpreting our results. Additional studies are needed to confirm these findings in the context of new SARS-CoV-2 variants and for new booster formulations.

## Introduction

Coronavirus Disease 2019 (COVID-19) vaccines reduce Severe Acute Respiratory Syndrome Coronavirus 2 (SARS-CoV-2) infections and symptom severity [1], yet breakthrough infections occur, especially with the Omicron (B.1.1.529) variant [2,3]. The effectiveness of Food and Drug Administration (FDA)-authorized or approved vaccines BNT162b2, mRNA-1273, and Ad26.COV2.S in preventing SARS-CoV-2 infections has dropped dramatically due to immune evasion and waning of vaccine-induced immunity over time [4–7]. The Omicron variant exhibits immune system escape as the result of several mutations [8,9]; this, and the high transmissibility of the Omicron variant, are leading to higher infection rates, strain on healthcare systems, and increased mortality [10,11]. With the emergence of new variants combined with waning immunity, the CDC recommends a booster 6 months after an initial mRNA vaccine series or 2 months after Ad26.COV2.S vaccination to prevent symptomatic and severe outcomes of COVID-19 [7,12]. The booster dose elicits an increase in antibody neutralization titers against the Omicron variant and causes affinity maturation leading to a better antibody response, maintaining long-term protection against severe COVID-19 outcomes [12–15].

While boosters are understood to be effective against severe disease, hospitalization [16], and symptomatic infections [12,17] resulting from the Omicron variant, limited information is available about their effectiveness against asymptomatic and mild symptomatic infections that may go unreported in the absence of asymptomatic surveillance testing. Asymptomatic and mild symptomatic infections play an important role in spreading SARS-CoV-2 [18]. Moreover, the clinical course of COVID-19 is understood to vary by age [19]; current estimates of booster effectiveness based on the general population may not apply to cohorts whose age distribution differs substantially from that of the general population.

We leveraged polymerase chain reaction (PCR) testing data derived from a SARS-CoV-2 surveillance program that required mandatory routine SARS-CoV-2 testing of students (through December 14, 2021) and departure tests before students left the Ithaca campus. During December 5 to December 31, 2021, there was an explosive increase in Omicron cases to the point where the Omicron variant (BA.1) became predominant at Cornell University’s Ithaca campus [20], and 1,926 SARS-CoV-2 infections were identified in a vaccinated population of students. We sought to estimate the effectiveness of COVID-19 boosters in reducing SARS-CoV-2 infections in the vaccinated population of students during this Omicron-predominant period.

## Methods

We used a retrospective cohort study to estimate the effectiveness of COVID-19 boosters in reducing SARS-CoV-2 infections. Specific to our context was a highly vaccinated study population, and a period where Omicron BA.1 was the dominant viral strain. We did not have an a priori protocol or statistical analysis plan. As detailed in Section A in S1 Appendix, our initial analysis [21] came as part of Cornell University’s institutional response to COVID-19 during an Omicron-driven outbreak among a highly vaccinated population. This plan was made after observing aggregate infection rates that compared those who had uploaded early proof of booster vaccination (less than 17% of those in the study population who would eventually provide documentation) against those who had not. We subsequently developed a study plan that modified two of the covariates in this analysis (adding sex and changing how primary vaccine series completion date was coded) because we hypothesized that they are confounders. We then made additional modifications in response to reviewer requests. As described in Section A in S1 Appendix, estimates of vaccine booster effectiveness were not sensitive to these changes in the analysis.

We utilized deidentified student data from Cornell University’s COVID-19 surveillance database. We included PCR-positive cases identified at Cornell surveillance testing sites, the campus student health service, and a sampling site operated by the local hospital system during the period of December 5, 2021 to December 31, 2021 (referred to as the “Omicron outbreak” or the study period), in which the Omicron variant (BA.1) was the predominant strain. Samples collected at Cornell surveillance sites and the local hospital’s sampling site were tested using EZ-SARS-CoV-2 Real-Time RT-PCR [22] within a two-stage Dorfman procedure [23] using pools of size 5. The assay targets two highly conserved regions of the SARS-CoV-2 N gene, and the Omicron variant is not expected to significantly affect test performance. Samples collected at the campus student health service were tested using the Cepheid Xpert Xpress SARS-CoV-2 Assay [24]. The FDA reports that the introduction of the Omicron variant does not appear to have resulted in a significant change in the test performance of this assay [25]. This work was completed as a part of Cornell’s institutional planning and preparedness, designated as exempt from IRB review by the Cornell IRB.

### Study context

In July 2020, as a part of its reopening strategy, Cornell implemented a robust COVID-19 surveillance testing program for students, faculty, and staff (1,637,394 PCR tests performed as of February 2, 2022). At the start of December 2021, 23,389 students were enrolled in full-time academic programs at Cornell’s Ithaca campus. Masks were required in all on-campus buildings. COVID-19 vaccination was required for all students (97% vaccinated), though medical or religious exemptions were granted. Boosters were encouraged (October 21, 2021) before being required (January 31, 2022). Through December 14, 2021, all undergraduates and professional program (veterinary, business, and law) students (*n =* 17,017) were enrolled in at-least-weekly mandatory PCR surveillance (compliance: 99.8% for surveillance tests scheduled between November 29, 2021 and December 14, 2021) using an anterior nasal swab sample. Approximately 86% of person-days included in the study (see the **Study population** section below) fell within this period. Students leaving the Ithaca campus before the end of the study period (82% of students in the study population indicated that they left before the end of the study period through a Cornell-managed checklist) were required to seek an additional test shortly before departure. Additionally, students were prompted to seek a PCR test if they were identified as a close contact of a case or if they developed COVID-like symptoms. Free PCR testing was available to any constituent of the university 7 days per week. Whole genome sequencing confirmed the presence of the Omicron variant (BA.1) in Cornell community COVID-19 surveillance samples collected on December 1, 2021; by December 11, 2021, Omicron was the predominant variant on the Ithaca campus [20].

### Study population

**Fig 1:**
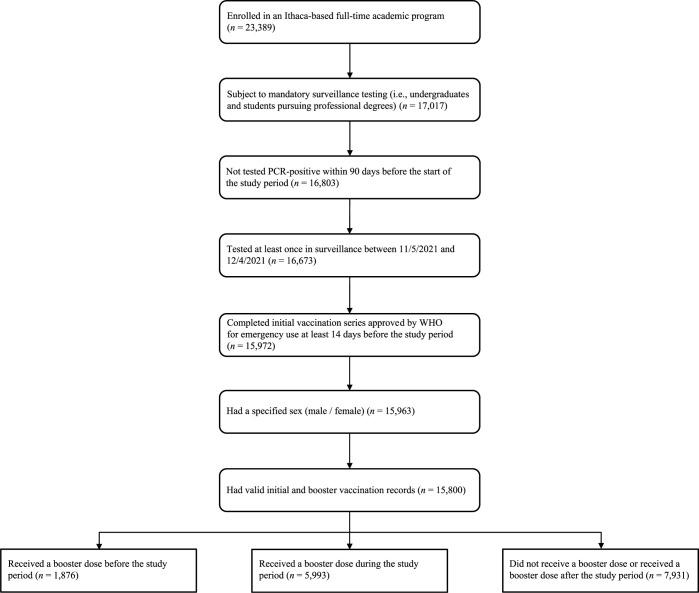
Enrollment of the study population. Students were included if they were enrolled in a full-time academic program based at Cornell’s Ithaca campus in Fall 2021, were subject to mandatory surveillance testing, had no positive SARS-CoV-2 PCR test within 90 days before the start of the study period, had at least one PCR test between November 5, 2021 and December 4, 2021, had a valid vaccination record indicating completion of WHO-approved initial vaccination series at least 14 days before the study period, and specified their sex in university records. PCR, polymerase chain reaction; SARS-CoV-2, Severe Acute Respiratory Syndrome Coronavirus 2; WHO, World Health Organization.

Our study included 15,800 Cornell students. Study inclusion criteria were as follows: being an active student at Cornell’s Ithaca campus enrolled in mandatory surveillance testing, being vaccinated with a vaccine approved by the World Health Organization (WHO) for emergency use at the time of the study (2 doses of BNT162b2, 2 doses of mRNA-1273, 1 dose of Ad26.COV2.S, or 2 doses of other WHO-approved vaccines), and having no positive SARS-CoV-2 PCR test within 90 days before the start of the study period (**Fig 1**). Students were required to upload a photo of a vaccine card documenting their booster status via an online form or request an exemption by February 1, 2022 [26]. According to vaccination records that were uploaded and validated by February 8, 2022, 11.9% of the study population (*n =* 1,876) received a booster before the study period, with the earliest booster dose administered on August 3, 2021. Our analysis does not distinguish the booster vaccine manufacturer, although this information is available in **Table B in S1 Appendix**. Students were excluded from the study population if they were not subject to mandatory surveillance testing, i.e., graduate students (*n =* 6,372), tested PCR-positive within 90 days before the start of the study period (*n =* 214), did not have at least one PCR surveillance test between November 5, 2021 and December 4, 2021 (*n =* 130), had not completed a WHO-approved initial vaccine series (*n* = 701), had an unspecified sex (*n* = 9), or had invalid vaccination records (*n* = 163). Students with no PCR test records during the study period (*n* = 285) were considered lost to follow-up.

### Statistical analysis

The primary outcome of our study was PCR-confirmed SARS-CoV-2 infection. To estimate the effectiveness of COVID-19 boosters against SARS-CoV-2 infections during this Omicron-predominant period, we calculated the person-days that each student contributed to the boosted and nonboosted population during the study period. The total number of person-days contributed by a student is the number of days between December 5, 2021 and either their final test date in the study period or their first PCR-positive test date, whichever comes first. Of the study population, 38,023 PCR tests were performed during the study period. Approximately 98.2% of the students (*n =* 15,515) had at least one PCR test (mean = 2.4 tests, median = 2.0 tests, SD = 1.2 tests), hence contributing at least one person-day (**Fig A in S1 Appendix**). We assume that booster vaccinations become effective 7 days after administration, based on results from a study of the Israeli general population [27]. As a result, each person-day has its booster status labeled as boosted (1) or control (0) based on whether the associated individual received their booster dose at least 7 days before that day (**Fig B in S1 Appendix**).

We performed a multivariable Poisson regression to estimate the effect of receiving a booster dose on having a positive PCR-based diagnosis during the study period. The unit of observation is an exposure period of an individual (measured in person days). An offset equal to log(person-days), i.e., the natural logarithm of the number of days in the exposure period, is added in the regression model for each exposure period. Each exposure period lasts until the end of the week (Week 0 is December 5 to December 11, etc.), a change in booster status (in which case a week is split into two exposure periods), or the person completes their last test in the study, whichever comes first. This model can be seen as a Cox proportional hazards analysis [28], in which it is assumed that exposure risk accrues at a constant rate within each exposure period. To account for possible within-individual correlation among observations over time (i.e., the risk of some individual contracting the disease is likely persistent over time due to individual behavior), we used generalized estimating equations (GEEs) [29,30] with an exchangeable correlation structure over weeks in the study period to obtain consistent coefficient estimates and robust variance estimates for the regression model.

We controlled for sex, student group (undergraduate or professional), fraternity/sorority participation (yes or no), varsity athletic team participation (yes or no), initial vaccine type (BNT162b2, mRNA-1273, Ad26.COV2.S, or other WHO-approved vaccines), initial vaccination series completion date (date of receiving 1 dose of Ad26.COV2.S or 2 doses of other WHO-approved vaccines, grouped by months from January 2021 to November 2021), and temporal effect during the study period (grouped by week). We also created univariable models for each of these covariates to obtain unadjusted incidence rate ratios. We included student group, fraternity/sorority participation, and athletic team participation as covariates because case investigation of data from before the study period suggested that these covariates explained much of the heterogeneity in the risk of infection across students [31]. Undergraduate students were divided into 3 subgroups based on fraternity/sorority and athletic team participation. Students who were in both fraternities/sororities and athletic teams were classified in the fraternity/sorority group. Thus, we had six student groups: (1) undergraduate fraternity/sorority participants; (2) undergraduate athletes not in Group 1; (3) undergraduates not in Groups 1 or 2; (4) law students; (5) postbaccalaureate business students; (6) veterinary students. Initial vaccination series completion date was included as a covariate to adjust for heterogeneous social behavior, inclination to receive vaccination, and waning vaccine immune response. The week during the study period was included as a categorical covariate to control for time-varying prevalence and the resulting risk of exposure. This mitigates bias that would have otherwise been created by temporal variation in the fraction of on-campus students boosted. We used a categorical covariate rather than a continuous one because prevalence did not change linearly with time. We did not include age as a covariate because there is little age variation in the study population (**Fig C in S1 Appendix**). The dependent variable was whether a student tests PCR-positive for COVID-19 in a particular exposure period. The regression model was

$$\begin{array}{l} \log(\lambda )=\log(\mathrm{exposure})+\beta_{0}+\beta_{1}*\mathrm{Booster}+\beta_{2}*\mathrm{Sex}\\ \;+\sum_{i=1}^{5}\gamma_{i}*\mathrm{Student}\;{\mathrm{Group}}_{i}\\ \;+\sum_{j=1}^{3}\alpha_{j}*\mathrm{Initial}\;\mathrm{Vaccine}\;{\mathrm{Type}}_{j}\\ \;+\sum_{k=1}^{10}\delta_{k}*\mathrm{Month}\;\mathrm{of}\;\mathrm{Initial}\;\mathrm{Vaccination}\;\mathrm{Series}\;{\mathrm{Completion}}_{k}\\ \;+\sum_{l=1}^{3}\sigma_{l}*\mathrm{Week}\;\mathrm{During}\;\mathrm{the}\;\mathrm{Study}\;{\mathrm{Period}}_{l} \end{array}$$

where *λ* is the incidence rate, and greek letters on the right-hand side of the specification are the regression coefficients. The *p-*values for the estimated coefficients and the 95% confidence intervals for the adjusted incidence rate ratios were adjusted using the Bonferroni correction [32].

To estimate the effectiveness of a booster dose, we used

$$booster\;effectiveness=1-adjusted\;incidence\;rate\;ratio\;(aIRR)=1-\exp\left(\beta_{1}\right).$$

We performed several robustness checks. To assess the effect of students leaving campus at the end of the semester and the accordingly paused mandatory surveillance, we performed a secondary GEE Poisson regression with the same specification as above, but for a shorter study period (from December 5, 2021 to December 18, 2021, the last day of exams).

To assess the possibility of bias remaining in our analysis despite the included controls, e.g., bias due to days since booster dose administration, we performed a GEE Poisson regression with multiple classes for the booster status (unboosted, 0 to 6 days after booster administration, ≥7 days after booster dose administration). We hypothesized that booster vaccination has negligible effect 0 to 6 days after administration.

We further performed a sensitivity analysis on the delay to boosted status, allowing person-days to count as boosted only after this delay after administration had elapsed, varying this delay from the day after booster administration (day 1) to 14 days (**Fig B in S1 Appendix**). We emphasize that varying this parameter only affects the booster status of person days from people that were boosted within or shortly before the start of the study period. We hypothesized that the shorter delays would result in smaller but still statistically significant booster effectiveness estimates as person-days from 0 to 6 days after booster administration would dilute the effect observed in ≥7 days after booster administration group. We expected to see similar estimates of booster effectiveness for all delays ≥7 days. We emphasize that this analysis is not designed to comment on how quickly boosters become effective, but instead only on the robustness of our main findings to assumptions about this delay parameter.

We also performed a logistic regression with GEE to assess the robustness of our conclusions to model specification. The unit of observation is a person-day. We assumed that booster vaccinations become effective 7 days after administration. The dependent variable is whether a student tests PCR-positive for SARS-CoV-2 on a particular day. The regression model was

$$\begin{array}{l} \log(\frac{p}{1-p})=\log(\mathrm{exposure})+\beta_{0}+\beta_{1}*\mathrm{Booster}+\beta_{2}*\mathrm{Sex}\\ \;+\sum_{i=1}^{5}\gamma_{i}*\mathrm{Student}\;\mathrm{Group}p_{i}\\ \;+\sum_{j=1}^{3}\alpha_{j}*\mathrm{Initial}\;\mathrm{Vaccine}\;{\mathrm{Type}}_{j}\\ \;+\sum_{k=1}^{10}\delta_{k}*\mathrm{Month}\;\mathrm{of}\;\mathrm{Initial}\;\mathrm{Vaccination}\;\mathrm{Series}\;{\mathrm{Completion}}_{k}\\ \;+\sum_{l=1}^{3}\sigma_{l}*\mathrm{Week}\;\mathrm{During}\;\mathrm{the}\;\mathrm{Study}\;{\mathrm{Period}}_{l} \end{array}$$

where *p* is the probability of infection, and greek letters on the right-hand side of the specification are the regression coefficients. This logistic model can be seen as a form of discrete time interval–censored survival model, in which a person’s endpoint is the first day on which they tested positive, and the right-censoring date is the date of their last negative test. As with the Poisson regression model, information bias is possible because testing did not occur every day. We approximated the incidence rate ratio using the adjusted odds ratio (aOR) when estimating the booster effectiveness.

A subset analysis of undergraduate students living on campus was performed to assess the robustness of our results to additional kinds of student clustering, i.e., residence, through which virus could spread. The description of this analysis is included in **Section B in S1 Appendix**.

All statistical analyses were performed in Python (V3.7.11), using the statsmodels package (V0.13.2). All code is publicly available (https://github.com/jiayuewan/booster_effectiveness). The data are protected by an institutional data requirement at Cornell that covers the use of data collected during COVID-19 surveillance and are maintained by Cornell University’s Office of the Vice President for Research and Innovation. These can be requested from that entity.

## Results

**Fig 2:**
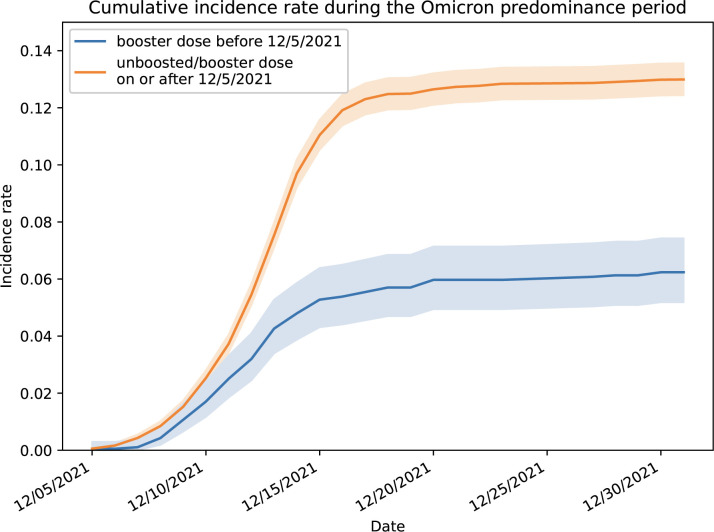
SARS-CoV-2 infection cumulative incidence rate (number of infections per person) and its 95% confidence interval during the study period, broken out by booster dose status.

Boosted students were more likely to be female, professional students, and have an earlier initial vaccine series than unboosted individuals (**Table 1**). During the study period, a total of 1,926 PCR-positive cases were identified and reported out of 15,800 students included in this analysis (overall infection risk of 12.2%). None of the cases required hospitalization. The overall infection risk among students that received a booster dose before December 5, 2021 is 6.2% (117 PCR-positives out of 1,876 boosted students), whereas the infection risk among students that did not receive a booster before December 5, 2021 is 13.0% (1,809 PCR-positives out of 13,924 students). The cumulative incidence among students who received a booster before December 5, 2021 was approximately half the cumulative incidence among students who were unboosted at that time (**Fig 2**). Similarly, the incidence rate was 0.6 per 100 person-days (127 PCR-positive cases/19,842 person-days) among students who had received the booster dose ≥7 days earlier, compared to 1.3 per 100 person-days (1,799 PCR-positive cases/135,214 person-days) among those who had not received the booster dose or had received the booster dose <7 days earlier.

**Table 1:** Distribution of person-day data by sex, student group, initial vaccination series completion date, initial vaccine type, and week in the study period.

	# unboosted person-days	# unboosted PCR-positive cases	Incidence rate per 100 person-days (unboosted)	# boosted person-days	# boosted PCR-positive cases	Incidence rate per 100 person days (boosted)	Total # person-days	Total # PCR-positive cases	Incidence rate per 100 person days (total)
	*n* (col %)	*n* (col %)		*n* (col %)	*n* (col %)		*n* (col %)	*n* (col %)	
**Sex**									
Female	72,195 (53%)	908 (50%)	1.3	12,034 (61%)	75 (59%)	0.6	84,229 (54%)	983 (51%)	1.2
Male	63,019 (47%)	891 (50%)	1.4	7,808 (39%)	52 (41%)	0.7	70,827 (46%)	943 (49%)	1.3
**Student group** ^**a**^									
UG-frat/sor	21,333 (16%)	517 (29%)	2.4	2,986 (15%)	35 (28%)	1.2	24,319 (16%)	552 (29%)	2.3
UG-athlete	7,629 (6%)	170 (9%)	2.2	781 (4%)	7 (6%)	0.9	8,410 (5%)	177 (9%)	2.1
UG-other	89,860 (66%)	985 (55%)	1.1	11,446 (58%)	65 (51%)	0.6	101,306 (65%)	1,050 (55%)	1.0
LA	6,166 (5%)	30 (2%)	0.5	1,734 (9%)	3 (2%)	0.2	7,900 (5%)	33 (2%)	0.4
GM	4,979 (4%)	84 (5%)	1.7	1,061 (5%)	15 (12%)	1.4	6,040 (4%)	99 (5%)	1.6
VM	5,247 (4%)	13 (1%)	0.2	1,834 (9%)	2 (2%)	0.1	7,081 (5%)	15 (1%)	0.2
**Month of initial vaccine series completion** ^**b**^									
January 2021	278 (0%)	5 (0%)	1.8	158 (1%)	1 (1%)	0.6	436 (0%)	6 (0%)	1.4
February 2021	1,926 (1%)	27 (2%)	1.4	1,185 (6%)	8 (6%)	0.7	3,111 (2%)	35 (2%)	1.1
March 2021	9,623 (7%)	184 (10%)	1.9	3,801 (19%)	34 (27%)	0.9	13,424 (9%)	218 (11%)	1.6
April 2021	41,897 (31%)	667 (37%)	1.6	9,006 (45%)	60 (47%)	0.7	50,903 (33%)	727 (38%)	1.4
May 2021	54,169 (40%)	646 (36%)	1.2	3,653 (18%)	21 (17%)	0.6	57,822 (37%)	667 (35%)	1.2
June 2021	10,883 (8%)	113 (6%)	1.0	1,095 (6%)	2 (2%)	0.2	11,978 (8%)	115 (6%)	1.0
July 2021	7,958 (6%)	77 (4%)	1.0	582 (3%)	0 (0%)	0.0	8,540 (6%)	77 (4%)	0.9
August 2021	5,301 (4%)	55 (3%)	1.0	160 (1%)	0 (0%)	0.0	5,461 (4%)	55 (3%)	1.0
September 2021	2,774 (2%)	21 (1%)	0.8	106 (1%)	0 (0%)	0.0	2,880 (2%)	21 (1%)	0.7
October 2021	392 (0%)	4 (0%)	1.0	72 (0%)	1 (1%)	1.4	464 (0%)	5 (0%)	1.1
November 2021	13 (0%)	0 (0%)	0.0	24 (0%)	0 (0%)	0.0	37 (0%)	0 (0%)	0.0
**Initial vaccine series type**									
BNT162b2	83,826 (62%)	1,179 (66%)	1.4	12,532 (63%)	83 (65%)	0.7	96,358 (62%)	1,262 (66%)	1.3
mRNA-1273	38,480 (28%)	481 (27%)	1.3	3,297 (17%)	24 (19%)	0.7	41,777 (27%)	505 (26%)	1.2
Ad26.COV2.S	6,234 (5%)	101 (6%)	1.6	1,186 (6%)	11 (9%)	0.9	7,420 (5%)	112 (6%)	1.5
Other WHO-approved vaccines	6,674 (5%)	38 (2%)	0.6	2,827 (14%)	9 (7%)	0.3	9,501 (6%)	47 (2%)	0.5
**Week in the study period**									
Week 0	89,480 (66%)	520 (29%)	0.6	11,256 (57%)	46 (36%)	0.4	100,736 (65%)	566 (29%)	0.6
Week 1	39,642 (29%)	1,211 (67%)	3.1	6,263 (32%)	68 (54%)	1.1	45,905 (30%)	1,279 (66%)	2.8
Week 2	4,614 (3%)	47 (3%)	1.0	1,524 (8%)	8 (6%)	0.5	6,138 (4%)	55 (3%)	0.9
Week 3	1,478 (1%)	21 (1%)	1.4	799 (4%)	5 (4%)	0.6	2,277 (1%)	26 (1%)	1.1
**Total**	135,214 (100%)	1,799 (100%)	1.3	19,842 (100%)	127 (100%)	0.6	155,056 (100%)	1,926 (100%)	1.2

^a^Descriptions for student group categories. UG-frat/sor: Undergraduate students affiliated with fraternities/sororities; UG-athlete: Undergraduate varsity athletes that have no affiliation with fraternities/sororities; UG-other: Other undergraduate students; LA: Professional students in the law school; GM: Postbaccalaureate professional students in the business school; VM: Professional students in the college of veterinary medicine.

^b^Initial vaccination series completion is the date of receiving 1 dose of Ad26.COV2.S or 2 doses of other WHO-approved vaccines.

The booster effectiveness estimate from the main GEE Poisson model is 56% (95% CI: [42%, 67%]). In addition to boosters, the incidence rate in our fitted GEE Poisson model depends on the student group and the date of completing the initial vaccine series (**Table 2**). The incidence rate was significantly lower among students who completed the initial vaccine series after May 1, 2021 compared with those completing the initial vaccine series earlier. During the study period, the incidence rate was significantly higher among undergraduate students participating in fraternity and sorority activities (aIRR 2.16 [1.84, 2.54]) or belonging to athletic teams (aIRR 2.02 [1.57, 2.60]), and among professional students at the business school (aIRR 1.64 [1.15, 2.32]), when compared with other undergraduates. Students vaccinated with Ad26.COV2.S had a higher risk of infection relative to other vaccines, but the difference was not statistically significant in our model.

**Table 2:** Summary of the full GEE Poisson regression model with covariates for sex, student group, initial vaccination series completion date, initial vaccine type, and week in the study period.

Variable	Unadjusted IRR^c^	Multivariable GEE Poisson model		
		*p-*value^d^	aIRR	95% CI for aIRR
**Booster (ref = 0)**				
1	0.47	<0.001	0.44	[0.33, 0.58]
**Sex (ref = Female)**				
Male	1.15	>0.99	1.02	[0.89, 1.18]
**Student group**^**a**^ **(ref = UG-other)**				
UG-frat/sor	2.20	<0.001	2.16	[1.84, 2.54]
UG-athlete	2.00	<0.001	2.02	[1.57, 2.60]
LA	0.41	<0.001	0.43	[0.25, 0.73]
GM	1.51	<0.001	1.64	[1.15, 2.32]
VM	0.21	<0.001	0.17	[0.08, 0.37]
**Month of initial vaccine series completion**^**b**^ **(ref = May 2021)**				
January 2021	1.15	>0.99	1.53	[0.44, 5.28]
February 2021	0.97	>0.99	1.23	[0.74, 2.07]
March 2021	1.40	<0.001	1.48	[1.15, 1.89]
April 2021	1.23	<0.001	1.30	[1.10, 1.55]
June 2021	0.82	>0.99	0.95	[0.69, 1.29]
July 2021	0.77	>0.99	0.89	[0.60, 1.31]
August 2021	0.87	>0.99	0.86	[0.56, 1.32]
September 2021	0.63	0.62	0.62	[0.32, 1.20]
October 2021	0.93	>0.99	1.02	[0.29, 3.66]
November 2021	0.00	<0.001	<0.001	[<0.001, <0.001]
**Initial vaccine type (ref = BNT162b2)**				
mRNA-1273	0.93	>0.99	0.97	[0.82, 1.15]
Ad26.COV2.S	1.16	>0.99	1.11	[0.82, 1.49]
Other WHO-approved vaccines	0.37	0.002	0.53	[0.33, 0.87]
**Week in the study period (ref = Week 0)**				
Week 1	4.89	<0.001	5.37	[4.61, 6.25]
Week 2	1.52	<0.001	2.57	[1.63, 4.07]
Week 3	1.90	<0.001	3.71	[1.96, 7.01]

^a^Descriptions for student group categories. UG-frat/sor: Undergraduate students affiliated with fraternities/sororities; UG-athlete: Undergraduate varsity athletes that have no affiliation with fraternities/sororities; UG-other: Other undergraduate students; LA: Professional students in the law school; GM: Postbaccalaureate professional students in the business school; VM: Professional students in the college of veterinary medicine.

^b^Initial vaccination series completion is the date of receiving 1 dose of Ad26.COV2.S or 2 doses of other WHO-approved vaccines.

^c^Unadjusted incidence rate ratios are computed from results of univariable GEE Poisson regression models.

^d^Adjusted using Bonferroni correction to address multiple comparisons.

aIRR, adjusted incidence rate ratio; GEE, generalized estimating equation; IRR, incidence rate ratio; WHO, World Health Organization.

To study the robustness to population size variation as students left campus at the end of the semester, we performed a GEE Poisson regression (**Table C in S1 Appendix**) for a shorter period (December 5, 2021 to December 18, 2021). This yields a similar booster effectiveness of 57% (95% CI: [42%, 68%]), suggesting that our estimate of booster effectiveness from the full regression model is robust to the effect of students leaving campus at the end of the semester and the accordingly paused mandatory surveillance. As an additional robustness check, we performed a GEE Poisson regression (**Table D in S1 Appendix**) with multiple classes for the booster status of a person-day (unboosted, 0 to 6 days after booster administration, ≥7 days after booster dose administration). As expected, a booster status corresponding to 0 to 6 days after booster dose administration was not found to have a statistically significant effect on PCR-diagnosed infection (*P* > 0.99) relative to being unboosted.

We further performed a sensitivity analysis to assess the impact of the assumed delay between booster administration and booster effectiveness. Although 5,993 students received their booster dose during the study period (**Fig D in S1 Appendix**), 5,210 of them received it after leaving the active surveillance program at the end of the semester. These students did not contribute any person-days to the study after their last surveillance test, so they contributed only unboosted person-days. For the 783 students who received a booster dose while in active surveillance, we varied this delay from 1 day to 14 days and calculated the booster effectiveness against infection using the full model (**Figs B and E in S1 Appendix**). The estimated booster effectiveness is consistently above 48% and is relatively flat after 7 days, suggesting that our estimate of booster effectiveness is robust to variation in the time required to mount an effective immune response after booster vaccination. Similarly, the incidence in the boosted population does not vary substantially as we change the assumed delay between booster administration and effectiveness (**Table E in S1 Appendix**).

In a separate sensitivity analysis using logistic regression with GEE (**Table F in S1 Appendix**), the estimated booster effectiveness is 57% (95% CI: [43%, 68%]). The estimate from the logistic regression with GEE is close to the estimate from the full GEE Poisson regression model.

In a subset analysis (**Section B in S1 Appendix**) on the undergraduate population living in on-campus housing, we did not observe a significant change in the estimated booster effectiveness on the subpopulation whether or not an additional covariate for housing building was included in the regression. This subset analysis justifies the use of our regression model without controlling for housing type on the full study population, given that housing information is only available for the on-campus undergraduates.

## Discussion

This study is one of the first community studies to quantify booster vaccine effectiveness from an actively surveilled population of young adults. Our study provides evidence that booster vaccinations significantly reduced infections in university settings during periods when the Omicron BA.1 variant was predominant, supporting the implementation of booster vaccination requirements to minimize community transmission.

In this retrospective analysis of SARS-CoV-2 tests performed at Cornell University’s Ithaca campus during a 27-day period when Omicron BA.1 was the predominant variant, the incidence of COVID-19 infections was approximately halved among participants vaccinated with a booster dose of a COVID-19 vaccine approved by WHO for emergency use when compared with vaccinated participants without a booster dose. The calculated booster effectiveness was 56% (95% CI: [42%, 67%]), which is slightly lower than a previously reported effectiveness against symptomatic COVID-like illness in adults (66%; 95% CI: [64%, 68%]) in the same time period [17]. Our estimate is similar to the reduction in cumulative incidence of reported infections associated with booster vaccination during an Omicron wave in Los Angeles County [33] and consistent with a pooled estimate of booster effectiveness (47%; 95% CI: [19%, 65%]) from a meta-analysis of studies completed during periods of Omicron predominance across the globe [34]. Importantly, our analysis includes both symptomatic and asymptomatic infections because the student population was under active surveillance, whereas none of the studies included in the meta-analysis examined a population under active surveillance [7,35–40]. Interestingly, between two studies on the effectiveness of a second booster dose, i.e., fourth vaccine dose [41,42], one that used data collected during active surveillance [42] also found lower effectiveness than one that did not [41]. Students vaccinated with Ad26.COV2.S had a higher risk of infection relative to other vaccines, similar to other studies [43], though the difference was not statistically significant, likely because a small number of students had Ad26.COV2.S initial doses (**Table 1, Table B in S1 Appendix**). We did not find a booster status corresponding to 0 to 6 days after booster dose administration to have a statistically significant effect on PCR-diagnosed infection relative to being unboosted. This could be because of an insufficient immune response to the booster within 6 days of administration, consistent with previous findings [22], or because of a lack of statistical power resulting from a small number of person days in the 0-to-6-day window after booster administration.

We observed lower incidence rates among students with more recent initial vaccinations, consistent with waning protection against SARS-CoV-2 infections observed within a few months of completing the initial vaccination series [44]. Months in which fewer individuals completed their initial vaccine series did not have a statistically significant effect, consistent with fewer data points resulting in less statistical power. Varsity athletes and students in fraternities/sororities at institutions of higher education may have more social contact than other undergraduates; thus, consistent with previous studies, we found that this population may be at higher risk for SARS-CoV-2 infection [31,45,46]. As in other higher-education institutions [46], contact tracing at Cornell identified fraternity/sorority gatherings as significant spreading events. Local case investigation efforts also pointed to additional events, including post-Thanksgiving break travel and a series of end-of-semester gatherings, as contributing to Omicron spread. With the emergence of highly transmissible variants, travel and social gatherings may put students at increased risk of SARS-CoV-2 infection.

Our Poisson regression analysis is similar to Bar-On and colleagues’ [47], a previous study of vaccine booster effectiveness against COVID-19. See also Bar-on and colleagues’ study [41] for another similar approach to estimating vaccine booster effectiveness against COVID-19. There are two common alternative statistical approaches to estimating vaccine effectiveness. The first approach is the emulated trial design (e.g., [48]), which matches subjects with the same covariates in an observational study to emulate a randomized trial. This design offers more robustness against misspecification of the functional form by which booster status and infection risk depend on covariates, but requires a larger dataset to enable matching simultaneously on all covariates. We did not have a sufficiently large dataset to match on all the covariates included in our model. A second alternative approach is the test-negative case–control design [17], which controls bias by including those individuals who sought testing, and improvements that model the propensity to test [49]. Since testing was mandatory and compliance was high, it is not necessary to model the propensity to test in this study population.

## Limitations and conclusion

Several limitations of our approach should be considered in interpreting our results. First, we did not have an a priori protocol or statistical analysis plan because the analysis was initially done for institutional research purposes, and some analysis choices were made after observing the data (see **Section A in S1 Appendix**). Thus, confidence intervals and hypothesis tests would be invalidated by correlation that could have been unintentionally introduced between the data and the choice of analysis plan. Second, a limitation of the Poisson regression is information bias due to the fact that individuals were not tested every day. Delays in reporting a positive test would cause the period of the delay to have a misclassified infection status, whether this period is within one exposure period or spans multiple exposure periods. Third, there may be additional confounding bias because, even after controlling for covariates, people who avoid risk could also be more likely to get a booster vaccination, which could result in the boosted group having decreased exposure to SARS-CoV-2. This would cause our regression analyses to overestimate vaccine effectiveness. Conversely, if people had engaged in more risky behavior in response to perceived protection offered by booster vaccination, our analyses would underestimate vaccine effectiveness. Fourth, our data do not allow distinguishing between booster doses and additional doses for immunocompromised individuals, or distinguishing between symptomatic and asymptomatic infections for confirmed PCR-positive cases. As mentioned earlier, not all samples were tested for S-gene dropout, and sequencing of every PCR-positive case was not performed. If boosters are more effective against the Delta variant than the Omicron variant, then Delta infections during the study period would lead our analysis to overestimate booster effectiveness against Omicron. Fifth, enough COVID-19 cases were observed to provide sufficient statistical power for moderately precise booster effectiveness estimates, but not to estimate how booster effectiveness varies with the manufacturer of the booster dose or the original vaccine. Most students were boosted with BNT162b2 in this study (**Table B in S1 Appendix**). Sixth, we did not include information on previous COVID-19 infections and had no information on the students’ underlying medical conditions or use of nonpharmaceutical interventions, although students were required to wear a mask (cloth, medical, or respirator) while inside campus buildings. This may lead to confounding bias if medical history differentially affected booster choice or if booster choice affected the use of nonpharmaceutical interventions. Seventh, gaps in time between a student’s last test and their departure from Ithaca (or the end of the study period, whichever came first) could lead to attrition bias. Eighth, although nearly 90% of positive samples tested for S-gene dropout during the study period exhibited this marker of the Omicron BA.1 variant [20], and sequencing confirmed the presence of the Omicron variant in all samples sequenced during this period, not all samples were tested for S-gene dropout, and sequencing of each PCR-positive case was not performed. Finally, not every individual who was boosted before or during the study period may have uploaded their vaccination record, which may have led to information bias towards the null.

In conclusion, our findings are consistent with the notion that booster vaccine doses, relative to being vaccinated with the initial series, are effective in reducing infections in young adults during Omicron-predominant periods, thereby helping universities and other institutions to remain open safely. In the 2 months subsequent to the period covered in this study, almost the entire Cornell student body, if eligible, received at least 1 booster dose.

## Supporting information

S1 STROBE Checklist STROBE Checklist. (DOCX)

S1 Appendix Supplementary Appendix. Section A. Analysis Timeline. Section B. Additional Subset Analysis. Table A. Summary of the GEE Poisson regression model for the on-campus undergraduate population with and without housing building as an additional covariate, and other covariates for sex, student group, initial vaccine series completion date, initial vaccine type, week during the study period. Table B. Distribution of booster dose type among students in the study population that received their booster dose before or during the study period, broken out by initial vaccination and booster dose type. Table C. Summary of the GEE Poisson regression model in the shortened study period (December 5, 2021 to December 18, 2021) with covariates for sex, student group, initial vaccine series completion date, initial vaccine type, and week during the study period. Table D. Summary of the GEE Poisson regression model with multiclass booster status (divided into unboosted, 0–6 days after booster dose administration, and ≥7 days after booster dose administration) and covariates for sex, student group, initial vaccine series completion date, initial vaccine type, and week during the study period. Table E. Number of unboosted and boosted person-days, PCR-positive cases, incidence rate with respect to different assumed delays for the booster to become effective after administration. Table F. Summary of the GEE logistic regression model with covariates for sex, student group, initial vaccine series completion date, initial vaccine type, and week during the study period. Fig A. Distribution of the number of person-days contributed by each student in the study population. Fig B. Allocation of person-days to the control and booster cohorts. Fig C. Age distribution of students in the study population. Fig D. Cumulative number of students receiving COVID-19 booster dose, over time. Fig E. Mean and 95% confidence interval for the booster effectiveness against infection during the Omicron predominance period, as we vary the assumed delay for the booster dose to become effective after booster administration
(DOCX)

## Abbreviations

aIRR: adjusted incidence rate ratio
aOR: adjusted odds ratio
COVID-19: Coronavirus Disease 2019
FDA: Food and Drug Administration
GEE: generalized estimating equation
PCR: polymerase chain reaction
SARS-CoV-2: Severe Acute Respiratory Syndrome Coronavirus 2
WHO: World Health Organization
