# Supplementary material for: Booster vaccination protection against SARS-CoV-2 infections in young adults during an Omicron BA.1-predominant period: A retrospective cohort study
Source: PLoS Med. 2023 Jan 10;20(1):e1004153. doi: 10.1371/journal.pmed.1004153 (PMC9876273; doi:10.1371/journal.pmed.1004153)
Supplement: S1 Appendix — Section A. Analysis Timeline. Section B. Additional Subset Analysis. Table A. Summary of the GEE Poisson regression model for the on-campus undergraduate population with and without housing building as an additional covariate, and other covariates for sex, student group, initial vaccine series completion date, initial vaccine type, week during the study period. Table B. Distribution of booster dose type among students in the study population that received their booster dose before or during the study period, broken out by initial vaccination and booster dose type. Table C. Summary of the GEE Poisson regression model in the shortened study period (December 5, 2021 to December 18, 2021) with covariates for sex, student group, initial vaccine series completion date, initial vaccine type, and week during the study period. Table D. Summary of the GEE Poisson regression model with multiclass booster status (divided into unboosted, 0–6 days after booster dose administration, and ≥7 days after booster dose administration) and covariates for sex, student group, initial vaccine series completion date, initial vaccine type, and week during the study period. Table E. Number of unboosted and boosted person-days, PCR-positive cases, incidence rate with respect to different assumed delays for the booster to become effective after administration. Table F. Summary of the GEE logistic regression model with covariates for sex, student group, initial vaccine series completion date, initial vaccine type, and week during the study period. Fig A. Distribution of the number of person-days contributed by each student in the study population. Fig B. Allocation of person-days to the control and booster cohorts. Fig C. Age distribution of students in the study population. Fig D. Cumulative number of students receiving COVID-19 booster dose, over time. Fig E. Mean and 95% confidence interval for the booster effectiveness against infection during the Omicron predominance period, as we vary the a [file pmed.1004153.s002.docx]

# **Supplementary Appendix**

| **Supplementary Sections** | |
| --- | --- |
| *Section A. Analysis Timeline* | 2 |
| *Section B. Additional Subset Analysis* | 5 |
| **Supplementary Tables** | |
| *Table A. Summary of the GEE Poisson regression model for the on-campus undergraduate population with and without housing building as an additional covariate, and other covariates for sex, student group, initial vaccine series completion date, initial vaccine type, week during the study period* | 6 |
| *Table B. Distribution of booster dose type among students in the study population that received their booster dose before or during the study period, broken out by initial vaccination and booster dose type* | 8 |
| *Table C. Summary of the GEE Poisson regression model in the shortened study period (Dec 5, 2021 to Dec 18, 2021) with covariates for sex, student group, initial vaccine series completion date, initial vaccine type, and week during the study period* | 9 |
| *Table D. Summary of the GEE Poisson regression model with multi-class booster status (divided into unboosted, 0-6 days after booster dose administration, and ≥ 7 days after booster dose administration) and covariates for sex, student group, initial vaccine series completion date, initial vaccine type, and week during the study period* | 10 |
| *Table E. Number of unboosted and boosted person-days, PCR-positive cases, incidence rate with respect to different assumed delays for the booster to become effective after administration* | 12 |
| *Table F. Summary of the GEE logistic regression model with covariates for sex, student group, initial vaccine series completion date, initial vaccine type, and week during the study period* | 13 |
| **Supplementary Figures** | |
| *Fig A. Distribution of the number of person-days contributed by each student in the study population* | 14 |
| *Fig B. Allocation of person-days to the control and booster cohorts* | 15 |
| *Fig C. Age distribution of students in the study population* | 16 |
| *Fig D. Cumulative number of students receiving COVID-19 booster dose, over time* | 17 |
| *Fig E. Mean and 95% confidence interval for the booster effectiveness against infection during the Omicron predominance period, as we vary the assumed delay for the booster dose to become effective after booster administration* | 18 |

# Section A. Analysis Timeline

In this section we describe how the analysis methodology appearing in this article was chosen and adapted during the reviewing process. This is provided to support understanding the potential for statistical bias from adjustments to the statistical analysis in response to the data.

Some of the authors (Wan, Frazier, Clarkberg, Henderson) first analyzed the data used in this paper in December 2021 and January 2022 as part of Cornell University’s institutional response to the pandemic. This analysis was done to understand the effect of booster vaccines to help plan interventions, e.g., the frequency of asymptomatic screening, for Cornell’s Spring 2022 semester and was made publicly available on Cornell’s website in early February 2022 [1].

The analysis began on December 17, 2021. Prompted by a hypothesis that booster doses would decrease the risk of infection, and that this would be important for planning for the Spring 2022 semester, Clarkberg calculated population size and counts of infections diagnosed after December 5, 2021 broken out by (1) whether the individual had provided to Cornell by that date proof of receiving a booster dose; (2) whether that person was an undergraduate, graduate/professional student, or non-student employee. At this time, only 1,239 students had uploaded documentation of having received a booster dose, and only 2,055 employees had uploaded documentation, as many would not upload their documentation until closer to Cornell’s deadline of January 31, 2022. These counts showed that those who had uploaded documentation of receiving a booster dose had a smaller rate of diagnosed infection after December 5, 2021. It was understood that there was the potential for bias from confounding and from the fact that documentation of booster doses was missing for most individuals.

The data was broken out by student status (undergraduate, grad/professional, employee) because past data collected in the institutional response showed that infection rates were very different among these groups. The date of December 5, 2021 was chosen as the start of the study period because this date was understood within those working in Cornell’s institutional response to the pandemic to be the beginning of the Omicron surge.

These counts were recomputed on December 21, 2021, by which point 2,148 students and 2,262 employees had uploaded documentation of having received a booster. As they did on December 17, these counts showed that those who had uploaded documentation of receiving a booster shot had a smaller rate of diagnosed infection during the study period. A member of the institutional response (Dr. Lisa Nishii) asked to check for confounding due to fraternity and sorority members both having behaviors that put them more at risk of infection and having been more likely to receive a booster dose. Counts showed a similar effect for both fraternity/sorority members and other undergraduates.

At this point, starting on December 20, 2021 and continuing through late December 2021, Wan and Frazier planned the analysis presented in the report referenced above [1]. This planning was done without accessing the available data on which individuals had been boosted, except as described in the paragraph above. Indeed, the data on booster status available to the authors at this time was less than 17% of the data that would ultimately be collected (2,148 is 17% of the 13,065 students in the study population, and the count of 2,148 includes students who were not in the study population).

This analysis used a logistic regression on person days similar to the sensitivity analysis presented in the **Methods** section with the regression specification

$infection \sim1+C(booster)+C(student group)+C(first dose date)+C(initial vaccine series type)$*.*

Compared to simple observations of counts, this analysis:

- Planned to use all of the available data on booster documentation. Most students analyzed in the analysis uploaded their documentation regarding booster vaccination after the analysis was planned, because Cornell announced a requirement after the study was planned requiring booster vaccinations with documentation provided by January 31, 2022.
- Focused only on students, because students had higher infection rates and so provided better statistical power and because they were the population of primary importance for public health planning with regard to the effect of booster vaccination on infection and its implications for surveillance testing, an important part of Cornell’s institutional response.
- Controlled for confounding using the student group covariate in the same way as we do elsewhere in this article. The use and definition of the student group covariate was chosen based on knowledge that this covariate explains a substantial amount of variation in infection risk at Cornell University; see, e.g., [2, 3].
- Controlled for confounding from the date and type of primary vaccination.
- Used logistic regression to control for multiple confounders and provide confidence intervals.

The analysis was identical to the logistic regression presented in the **Methods** section, except that:

- It assumed independent errors and used maximum likelihood estimation rather than assuming clustered errors and using generalized estimation equations.
- It did not control for the week when the student was diagnosed or the student’s sex.
- Rather than controlling for the date of primary vaccination using the month of completion of the last dose, as in the **Methods** section, it used a coarse discretization of when the first dose in the initial vaccine series was administered.
- Data on booster status was only available for students and employees who had uploaded their information at the time the analysis was posted. A substantial number of additional students uploaded records of having received a booster dose after this first analysis was completed to comply with Cornell’s decision to mandate boosters.

That report also included a sensitivity analysis, which was planned at the same time as the primary analysis (late December 2021), and which varied the assumed number of days between administration of a booster dose and when it is considered to become effective.

After this analysis was complete, we then planned our analysis for submission as a research article. For initial submission, we maintained a logistic regression over person days using independent errors and made the following modifications:

- We added sex as a covariate. We did this because one of the co-authors not associated with the original analysis (Lee) hypothesized that sex could cause confounding and because this is a common covariate controlled for in observational studies. This hypothesis was made without examining the data. We did not include sex as a covariate in our original analysis because experience with data from before the study period consistently suggested that sex is not predictive of infection risk among the student population at Cornell.
- We modified the covariate used to control for the date of vaccination to be a categorical variable indicating whether the date when a person was considered fully vaccinated (i.e., 14 days after completion of a primary vaccination series) was in (1) before May 2021 (23% of boosted individuals) (2) in May 2021 (49%) (3) in June 2021 (15%) or (4) after June 2021 (14%). We used this categorization because we were concerned about using a finer categorization for periods before May 2021 and after June 2021 when the fraction of the study population boosted in each month was small. Many students received initial vaccinations in May or June when vaccines first became widely available.

We also presented a sensitivity analysis using Poisson regression that used the same set of covariates. Each individual provided one datapoint if they were considered boosted during the whole study period, and two datapoints if they were considered unboosted at the beginning of the study period and boosted later on in the study period. Datapoints were modeled as independent.

Then, during the reviewing process and at the suggestion of reviewers and editors, we made the following changes to our main analysis:

- The Poisson regression, formerly a sensitivity analysis, became the main analysis. It was also changed to include a separate datapoint for each week, as discussed in the main paper. The logistic regression was retained for sensitivity analysis.
- We included the week during the study period as a covariate. This was added to control for confounding due to changes in infection risk over time, which could have been correlated with the fraction of the population boosted in that week.
- We added “other vaccines” in addition to the three initial vaccine series types we had considered previously (i.e., BNT162b2, mRNA-1273, and Ad26.COV2.S).
- We changed the covariate used to control for when the primary vaccine series was completed to include a categorical variable for each month. This was done to address a concern from a reviewer that the previous categorization was ad hoc.
- We moved from assuming independent errors to using generalized estimating equations to control for within-individual correlation in infection risk that could arise from behavior that is either persistently less risky or more risky than the general population.

We also conducted several sensitivity analyses to respond to questions from the reviewing team that are presented in the **Methods** section and **Section B in S1 Appendix**. We also conducted a sensitivity analysis including the manufacturer of the booster, which was not included in the article because we hypothesized before conducting the analysis that the sample size would be too small to estimate differences in booster effectiveness between vaccine manufacturers and because the analysis, once conducted, confirmed this hypothesis.

Estimates of vaccine booster effectiveness were not sensitive to these changes in the analysis. The analysis in the institutional report [1] estimated that a booster dose reduces the odds of having a PCR-detected SARS-CoV-2 infection relative to an initial vaccination series by 54% (95% confidence interval [45%, 62%], P <0.001). The analysis in the first submission as a research article estimated that a booster dose reduces the odds of infection by 52% (95% confidence interval [37%, 64%], P<0.001). For comparison, after incorporating comments from the reviewing team, our main analysis estimates that a booster dose further reduces the rate of infection relative to an initial vaccination series by 56% (95% confidence interval [42%, 67%], P <0.001).

# Section B. Additional Subset Analysis

At the suggestion of the reviewers, to assess the robustness of our regression results to additional kinds of student clustering, we performed a subset analysis on the undergraduates living in on-campus housing, including the building in which they lived as an additional covariate.

We performed a GEE Poisson regression analysis (**Table A in S1 Appendix**) on the on-campus undergraduate population (*n* = 6,570) whose housing information is available. We added the housing building in which they lived at the beginning of the study period as an additional covariate in the regression model.

When the covariate for housing building was included, we obtained an estimated booster effectiveness of 53% (95% CI: [16%, 74%]) (**Table A in S1 Appendix**). This estimate remains close to the estimate from the full model using GEE Poisson regression (i.e., 56%), but has a wider confidence interval due to the reduced study population. All but one of the *p*-values associated with housing buildings were found statistically insignificant at the 0.05 level, indicating insufficient evidence of confounding in infections due to on-campus housing. (The *p*-values and confidence intervals for the covariates associated with housing buildings are concealed here due to institutional concern of being able to recover building names from their statistics.)

To assess the robustness of our subset analysis, we performed a second GEE Poisson regression (**Table A in S1 Appendix**) on the same on-campus undergraduate population, without controlling for the housing building. This yields an estimated booster effectiveness of 51% (95% CI: [18%, 71%]). We observe that whether or not housing building was included as an additional covariate did not significantly change the regression result on the same subpopulation. This justifies the use of our regression model without controlling for housing building on the full study population, given that housing information is only available for the on-campus undergraduates.

# Table A. Summary of the GEE Poisson regression model for the on-campus undergraduate population with and without housing building as an additional covariate, and other covariates for sex, student group, initial vaccine series completion date, initial vaccine type, and week during the study period.

| **Variable** | **Multivariable GEE Poisson model (on-campus undergraduate population)** | | | | | |
| --- | --- | --- | --- | --- | --- | --- |
|  | **Adjusting for housing building** | | | **Without adjusting for housing building** | | |
|  | ***p*-value^c^** | **Adjusted incidence rate ratio (aIRR)** | **95% CI for aIRR** | ***p*-value^c^** | **Adjusted incidence rate ratio (aIRR)** | **95% CI for aIRR** |
| **Booster (ref = 0)** |  |  |  |  |  |  |
| 1 | <0.001 | 0.47 | [0.26, 0.84] | <0.001 | 0.49 | [0.29, 0.82] |
| **Sex (ref = Female)** |  |  |  |  |  |  |
| Male | >0.99 | 1.03 | [0.80, 1.33] | >0.99 | 1.00 | [0.79, 1.26] |
| **Student group^a^ (ref = UG-other)** |  |  |  |  |  |  |
| UG-frat/sor | <0.001 | 2.19 | [1.58, 3.03] | <0.001 | 2.27 | [1.70, 3.04] |
| UG-athlete | <0.001 | 2.18 | [1.30, 3.64] | <0.001 | 2.17 | [1.38, 3.40] |
| **Month of initial vaccine series completion^b^ (ref = May 2021)** |  |  |  |  |  |  |
| January 2021 | >0.99 | 2.88 | [0.57, 14.58] | >0.99 | 2.60 | [0.52, 13.13] |
| February 2021 | >0.99 | 0.94 | [0.30, 2.92] | >0.99 | 0.91 | [0.32, 2.64] |
| March 2021 | >0.99 | 1.28 | [0.73, 2.25] | >0.99 | 1.29 | [0.77, 2.15] |
| April 2021 | >0.99 | 1.14 | [0.84, 1.55] | >0.99 | 1.15 | [0.86, 1.52] |
| June 2021 | >0.99 | 0.97 | [0.60, 1.58] | >0.99 | 0.94 | [0.60, 1.47] |
| July 2021 | >0.99 | 1.08 | [0.61, 1.92] | >0.99 | 1.07 | [0.63, 1.81] |
| August 2021 | >0.99 | 1.12 | [0.58, 2.14] | >0.99 | 1.13 | [0.62, 2.04] |
| September 2021 | >0.99 | 0.80 | [0.32, 2.00] | >0.99 | 0.81 | [0.35, 1.88] |
| October 2021 | >0.99 | 1.47 | [0.31, 7.09] | >0.99 | 1.39 | [0.31, 6.32] |
| November 2021 | <0.001 | <0.001 | [<0.001, <0.001] | <0.001 | <0.001 | [<0.001, <0.001] |
| **Initial vaccine type (ref = BNT162b2)** |  |  |  |  |  |  |
| mRNA-1273 | >0.99 | 0.88 | [0.64, 1.22] | 0.73 | 0.82 | [0.61, 1.09] |
| Ad26.COV2.S | >0.99 | 0.92 | [0.49, 1.71] | >0.99 | 0.88 | [0.50, 1.56] |
| Other WHO-approved vaccines | 0.950 | 0.54 | [0.23, 1.28] | 0.180 | 0.51 | [0.23, 1.11] |
| **Week in the study period (ref = Week 0)** |  |  |  |  |  |  |
| Week 1 | <0.001 | 6.22 | [4.71, 8.20] | <0.001 | 6.13 | [4.75, 7.92] |
| Week 2 | >0.99 | 1.43 | [0.20, 10.48] | >0.99 | 1.28 | [0.21, 8.03] |
| Week 3 | 0.36 | 4.39 | [0.72, 26.83] | 0.41 | 3.77 | [0.67, 21.27] |

^a^ Descriptions for student group categories. UG-frat/sor: Undergraduate students affiliated with fraternities/sororities; UG-athlete: Undergraduate varsity athletes that have no affiliation with fraternities/sororities; UG-other: Other undergraduate students; LA: Professional students in the law school; GM: Postbaccalaureate professional students in the business school; VM: Professional students in the college of veterinary medicine.

^b^ Initial vaccination series completion is the date of receiving 1 dose of Ad26.COV2.S or 2 doses of other WHO-approved vaccines.

^c^ Adjusted using Bonferroni correction to address multiple comparisons.Table B. Distribution of booster dose type among students in the study population that received their booster dose before or during the study period, broken out by initial vaccination and booster dose type.

|  | **Initial vaccination type** | | | |
| --- | --- | --- | --- | --- |
|  | BNT162b2 | mRNA-1273 | Ad26.COV2.S | Other vaccines |
|  | *n* (col %) | *n* (col %) | *n* (col %) | *n* (col %) |
| **Booster dose type** |  |  |  |  |
| BNT162b2 | 4,262 (42%) | 454 (11%) | 168 (21%) | 207 (29%) |
| mRNA-1273 | 878 (9%) | 1,644 (39%) | 129 (16%) | 84 (12%) |
| Ad26.COV2.S | 8 (0%) | 5 (0%) | 27 (3%) | 0 (0%) |
| Other vaccines^a^ | 1 (0%) | 0 (0%) | 0 (0%) | 2 (0%) |
| Unboosted or boosted after the study period | 4,918 (49%) | 2,111 (50%) | 491 (60%) | 411 (58%) |
| **Total** | 10,067 (100%) | 4,214 (100%) | 815 (100%) | 704 (100%) |

^a^ Other vaccines approved by WHO for emergency use.

# Table C. Summary of the GEE Poisson regression model in the shortened study period (Dec 5, 2021 to Dec 18, 2021) with covariates for sex, student group, initial vaccine series completion date, initial vaccine type, and week during the study period.

| **Variable** | **Multivariable GEE Poisson model**  **(shortened study period)** | | |
| --- | --- | --- | --- |
|  | ***p*-value^c^** | **Adjusted incidence rate ratio (aIRR)** | **95% CI for aIRR** |
| **Booster (ref = 0)** |  |  |  |
| 1 | <0.001 | 0.43 | [0.32, 0.58] |
| **Sex (ref = Female)** |  |  |  |
| Male | >0.99 | 1.00 | [0.87, 1.16] |
| **Student group^a^ (ref = UG-other)** |  |  |  |
| UG-frat/sor | <0.001 | 2.14 | [1.82, 2.51] |
| UG-athlete | <0.001 | 2.10 | [1.64, 2.70] |
| LA | <0.001 | 0.32 | [0.16, 0.62] |
| GM | <0.001 | 1.79 | [1.26, 2.55] |
| VM | <0.001 | 0.10 | [0.03, 0.32] |
| **Month of initial vaccine series completion^b^ (ref = May 2021)** |  |  |  |
| January 2021 | >0.99 | 1.72 | [0.52, 5.70] |
| February 2021 | >0.99 | 1.27 | [0.76, 2.13] |
| March 2021 | <0.001 | 1.52 | [1.19, 1.95] |
| April 2021 | <0.001 | 1.30 | [1.09, 1.54] |
| June 2021 | >0.99 | 0.97 | [0.71, 1.32] |
| July 2021 | >0.99 | 0.88 | [0.59, 1.32] |
| August 2021 | >0.99 | 0.78 | [0.49, 1.25] |
| September 2021 | 0.37 | 0.55 | [0.26, 1.18] |
| October 2021 | >0.99 | 1.10 | [0.31, 3.84] |
| November 2021 | <0.001 | <0.001 | [<0.001, <0.001] |
| **Initial vaccine type (ref = BNT162b2)** |  |  |  |
| mRNA-1273 | >0.99 | 0.96 | [0.81, 1.14] |
| Ad26.COV2.S | >0.99 | 1.08 | [0.80, 1.46] |
| Other WHO-approved vaccines | 0.006 | 0.53 | [0.31, 0.90] |
| **Week in the study period (ref = Week 0)** |  |  |  |
| Week 1 | <0.001 | 5.38 | [4.62, 6.26] |

^a^ Descriptions for student group categories. UG-frat/sor: Undergraduate students affiliated with fraternities/sororities; UG-athlete: Undergraduate varsity athletes that have no affiliation with fraternities/sororities; UG-other: Other undergraduate students; LA: Professional students in the law school; GM: Postbaccalaureate professional students in the business school; VM: Professional students in the college of veterinary medicine.

^b^ Initial vaccination series completion is the date of receiving 1 dose of Ad26.COV2.S or 2 doses of other WHO-approved vaccines.

^c^ Adjusted using Bonferroni correction to address multiple comparisons.

# Table D. Summary of the GEE Poisson regression model with multi-class booster status (divided into unboosted, 0-6 days after booster dose administration, and ≥ 7 days after booster dose administration) and covariates for sex, student group, initial vaccine series completion date, initial vaccine type, and week during the study period.

| **Variable** | **Multivariable GEE Poisson model**  **(multi-class booster status)** | | |
| --- | --- | --- | --- |
|  | ***p*-value^c^** | **Adjusted incidence rate ratio (aIRR)** | **95% CI for aIRR** |
| **Booster (ref = Unboosted)** |  |  |  |
| 0 - 6 days after booster dose administration | >0.99 | 0.84 | [0.56, 1.26] |
| ≥ 7 days after booster dose administration | <0.001 | 0.43 | [0.32, 0.58] |
| **Sex (ref = Female)** |  |  |  |
| Male | >0.99 | 1.02 | [0.89, 1.18] |
| **Student group^a^ (ref = UG-other)** |  |  |  |
| UG-frat/sor | <0.001 | 2.16 | [1.83, 2.54] |
| UG-athlete | <0.001 | 2.03 | [1.57, 2.61] |
| LA | <0.001 | 0.43 | [0.25, 0.73] |
| GM | <0.001 | 1.65 | [1.16, 2.34] |
| VM | <0.001 | 0.17 | [0.08, 0.37] |
| **Month of initial vaccine series completion^b^**  **(ref = May 2021)** |  |  |  |
| January 2021 | >0.99 | 1.56 | [0.45, 5.43] |
| February 2021 | >0.99 | 1.24 | [0.74, 2.09] |
| March 2021 | <0.001 | 1.49 | [1.16, 1.91] |
| April 2021 | <0.001 | 1.31 | [1.10, 1.55] |
| June 2021 | >0.99 | 0.94 | [0.69, 1.29] |
| July 2021 | >0.99 | 0.88 | [0.60, 1.30] |
| August 2021 | >0.99 | 0.85 | [0.55, 1.32] |
| September 2021 | 0.59 | 0.62 | [0.32, 1.19] |
| October 2021 | >0.99 | 1.02 | [0.28, 3.66] |
| November 2021 | <0.001 | <0.001 | [<0.001, <0.001] |
| **Initial vaccine type (ref = BNT162b2)** |  |  |  |
| mRNA-1273 | >0.99 | 0.98 | [0.82, 1.16] |
| Ad26.COV2.S | >0.99 | 1.11 | [0.82, 1.50] |
| Other WHO-approved vaccines | 0.001 | 0.53 | [0.33, 0.87] |
| **Week in the study period (ref = Week 0)** |  |  |  |
| Week 1 | <0.001 | 5.39 | [4.62, 6.28] |
| Week 2 | <0.001 | 2.62 | [1.65, 4.16] |
| Week 3 | <0.001 | 3.77 | [1.99, 7.15] |

^a^ Descriptions for student group categories. UG-frat/sor: Undergraduate students affiliated with fraternities/sororities; UG-athlete: Undergraduate varsity athletes that have no affiliation with fraternities/sororities; UG-other: Other undergraduate students; LA: Professional students in the law school; GM: Postbaccalaureate professional students in the business school; VM: Professional students in the college of veterinary medicine.

^b^ Initial vaccination series completion is the date of receiving 1 dose of Ad26.COV2.S or 2 doses of other WHO-approved vaccines.

^c^ Adjusted using Bonferroni correction to address multiple comparisons.

# Table E. Number of unboosted and boosted person-days, PCR-positive cases, incidence rate with respect to different assumed delays for the booster to become effective after administration.

| **Delay (days)** | **# unboosted person-days** | **# unboosted PCR-positive cases** | **Incidence rate per 100 person-days (unboosted)** | **# boosted person-days** | **# boosted PCR-positive cases** | **Incidence rate per 100 person-days (boosted)** |
| --- | --- | --- | --- | --- | --- | --- |
| 1 | 131,474 | 1,740 | 1.32 | 23,582 | 186 | 0.79 |
| 2 | 132,184 | 1,750 | 1.32 | 22,872 | 176 | 0.77 |
| 3 | 132,872 | 1,762 | 1.33 | 22,184 | 164 | 0.74 |
| 4 | 133,520 | 1,773 | 1.33 | 21,536 | 153 | 0.71 |
| 5 | 134,127 | 1,779 | 1.33 | 20,929 | 147 | 0.70 |
| 6 | 134,700 | 1,787 | 1.33 | 20,356 | 139 | 0.68 |
| 7 | 135,214 | 1,799 | 1.33 | 19,842 | 127 | 0.64 |
| 8 | 135,679 | 1,802 | 1.33 | 19,377 | 124 | 0.64 |
| 9 | 136,206 | 1,808 | 1.33 | 18,850 | 118 | 0.63 |
| 10 | 136,872 | 1,813 | 1.32 | 18,184 | 113 | 0.62 |
| 11 | 137,485 | 1,816 | 1.32 | 17,571 | 110 | 0.63 |
| 12 | 138,311 | 1,818 | 1.31 | 16,745 | 108 | 0.64 |
| 13 | 139,185 | 1,821 | 1.31 | 15,871 | 105 | 0.66 |
| 14 | 140,072 | 1,827 | 1.30 | 14,984 | 99 | 0.66 |

#

# Table F. Summary of the GEE logistic regression model with covariates for sex, student group, initial vaccine series completion date, initial vaccine type, and week during the study period.

| **Variable** | **Multivariable GEE logistic model** | | |
| --- | --- | --- | --- |
|  | ***p*-value^c^** | **Adjusted odds ratio (aOR)** | **95% CI for aOR** |
| **Booster (ref = 0)** |  |  |  |
| 1 | <0.001 | 0.43 | [0.32, 0.57] |
| **Sex (ref = Female)** |  |  |  |
| Male | >0.99 | 1.02 | [0.89, 1.18] |
| **Student group^a^ (ref = UG-other)** |  |  |  |
| UG-frat/sor | <0.001 | 2.21 | [1.87, 2.62] |
| UG-athlete | <0.001 | 2.07 | [1.59, 2.69] |
| LA | <0.001 | 0.42 | [0.25, 0.72] |
| GM | <0.001 | 1.66 | [1.16, 2.37] |
| VM | <0.001 | 0.16 | [0.07, 0.36] |
| **Month of initial vaccine series completion^b^ (ref = May 2021)** |  |  |  |
| January 2021 | >0.99 | 1.55 | [0.43, 5.57] |
| February 2021 | >0.99 | 1.24 | [0.73, 2.11] |
| March 2021 | 0.001 | 1.49 | [1.16, 1.93] |
| April 2021 | <0.001 | 1.31 | [1.10, 1.57] |
| June 2021 | >0.99 | 0.95 | [0.69, 1.30] |
| July 2021 | >0.99 | 0.88 | [0.59, 1.31] |
| August 2021 | >0.99 | 0.85 | [0.55, 1.33] |
| September 2021 | 0.58 | 0.62 | [0.32, 1.19] |
| October 2021 | >0.99 | 1.03 | [0.28, 3.77] |
| November 2021 | <0.001 | <0.001 | [<0.001, <0.001] |
| **Initial vaccine type (ref = BNT162b2)** |  |  |  |
| mRNA-1273 | >0.99 | 0.97 | [0.82, 1.16] |
| Ad26.COV2.S | >0.99 | 1.11 | [0.81, 1.51] |
| Other WHO-approved vaccines | 0.001 | 0.53 | [0.32, 0.86] |
| **Week in the study period (ref = Week 0)** |  |  |  |
| Week 1 | <0.001 | 5.58 | [4.77, 6.52] |
| Week 2 | <0.001 | 2.64 | [1.66, 4.19] |
| Week 3 | <0.001 | 3.88 | [2.04, 7.36] |

^a^ Descriptions for student group categories. UG-frat/sor: Undergraduate students affiliated with fraternities/sororities; UG-athlete: Undergraduate varsity athletes that have no affiliation with fraternities/sororities; UG-other: Other undergraduate students; LA: Professional students in the law school; GM: Postbaccalaureate rofessional students in the business school; VM: Professional students in the college of veterinary medicine.

^b^ Initial vaccination series completion is the date of receiving 1 dose of Ad26.COV2.S or 2 doses of other WHO-approved vaccines.

# ^c^ Adjusted using Bonferroni correction to address multiple comparisons.
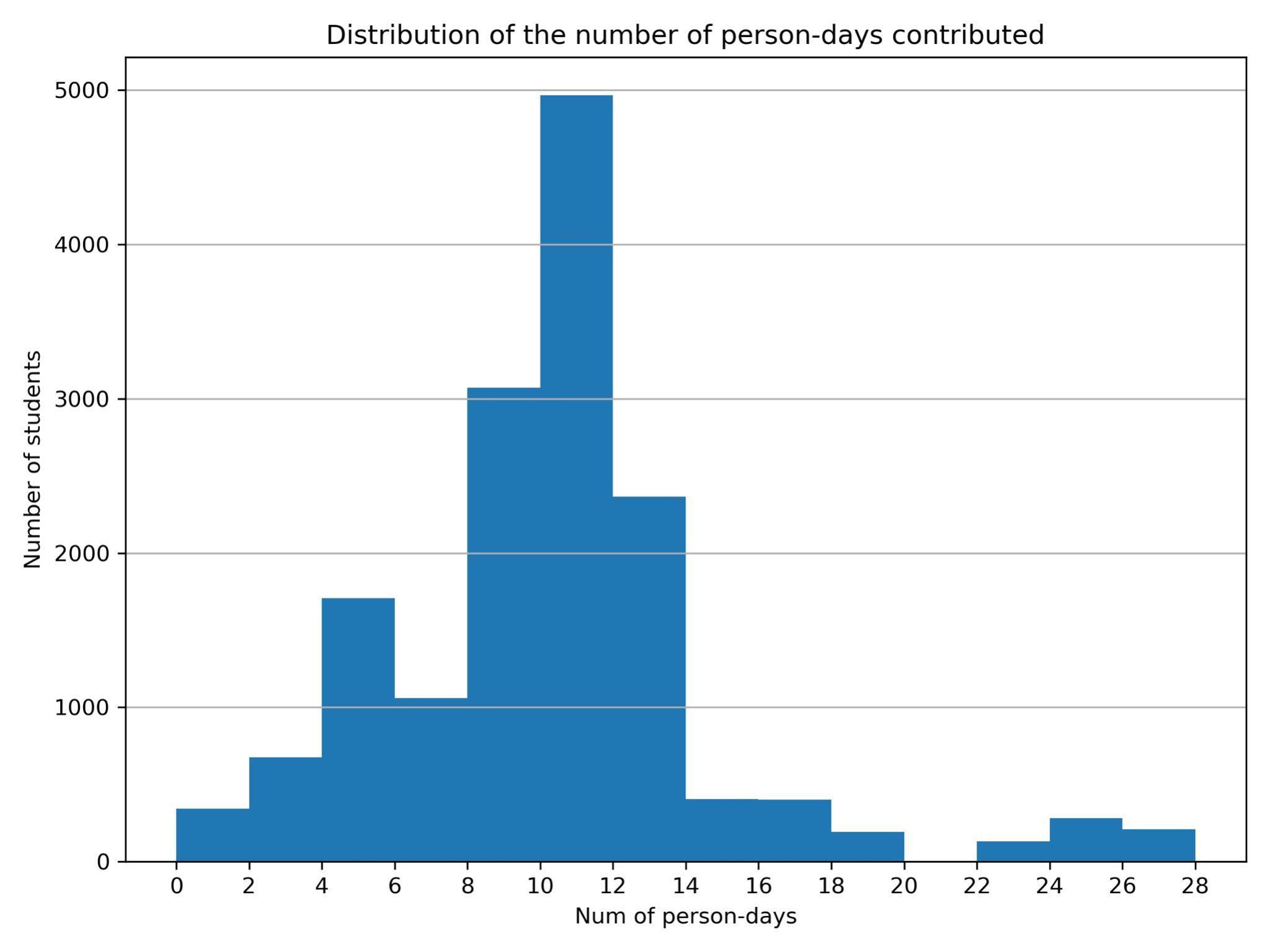
Fig A. Distribution of the number of person-days contributed by each student in the study population (n = 15,800). Mean = 9.8 person-days; median = 10 person-days; interquartile range = 4 person-days.

#
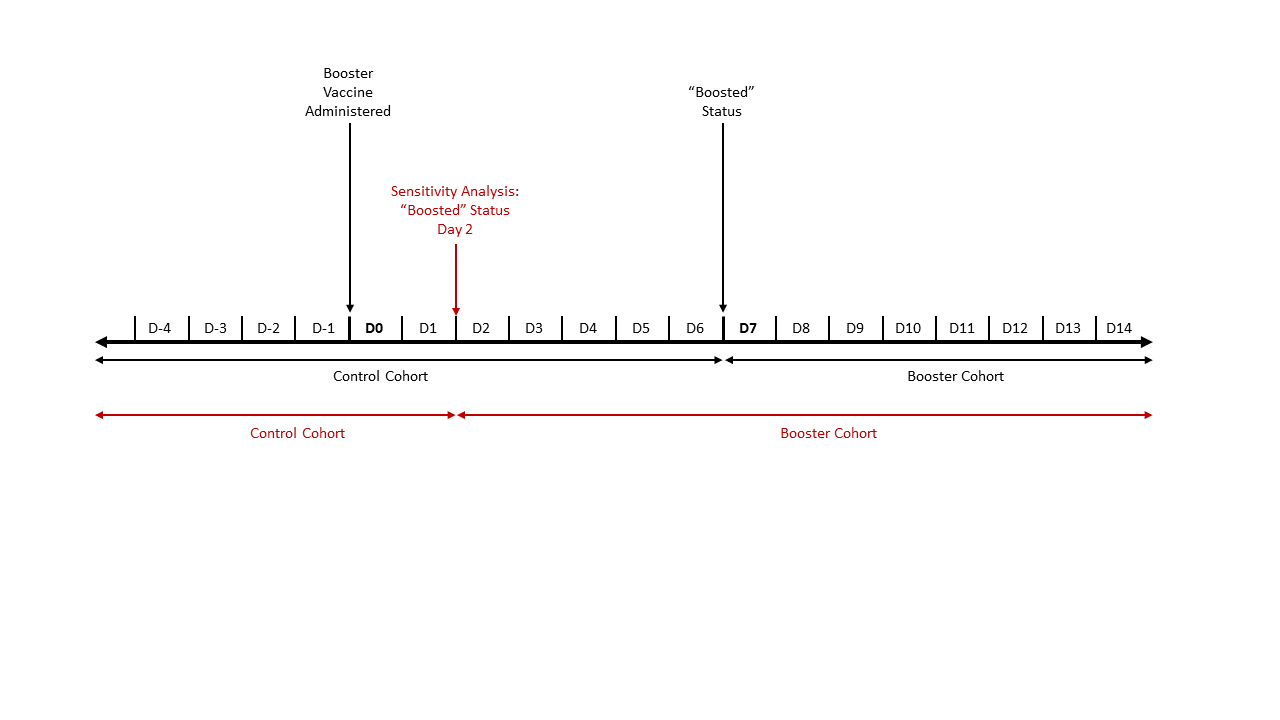
Fig B. Allocation of person-days to the control and booster cohorts. The black timeline shows the main analysis, with students having a “boosted” status 7 days after SARS-CoV-2 booster vaccine administration. The red timeline shows one example of our sensitivity analysis varying the time to “boosted” status, with students in this example achieving a “boosted” status 2 days after booster vaccine administration.

#
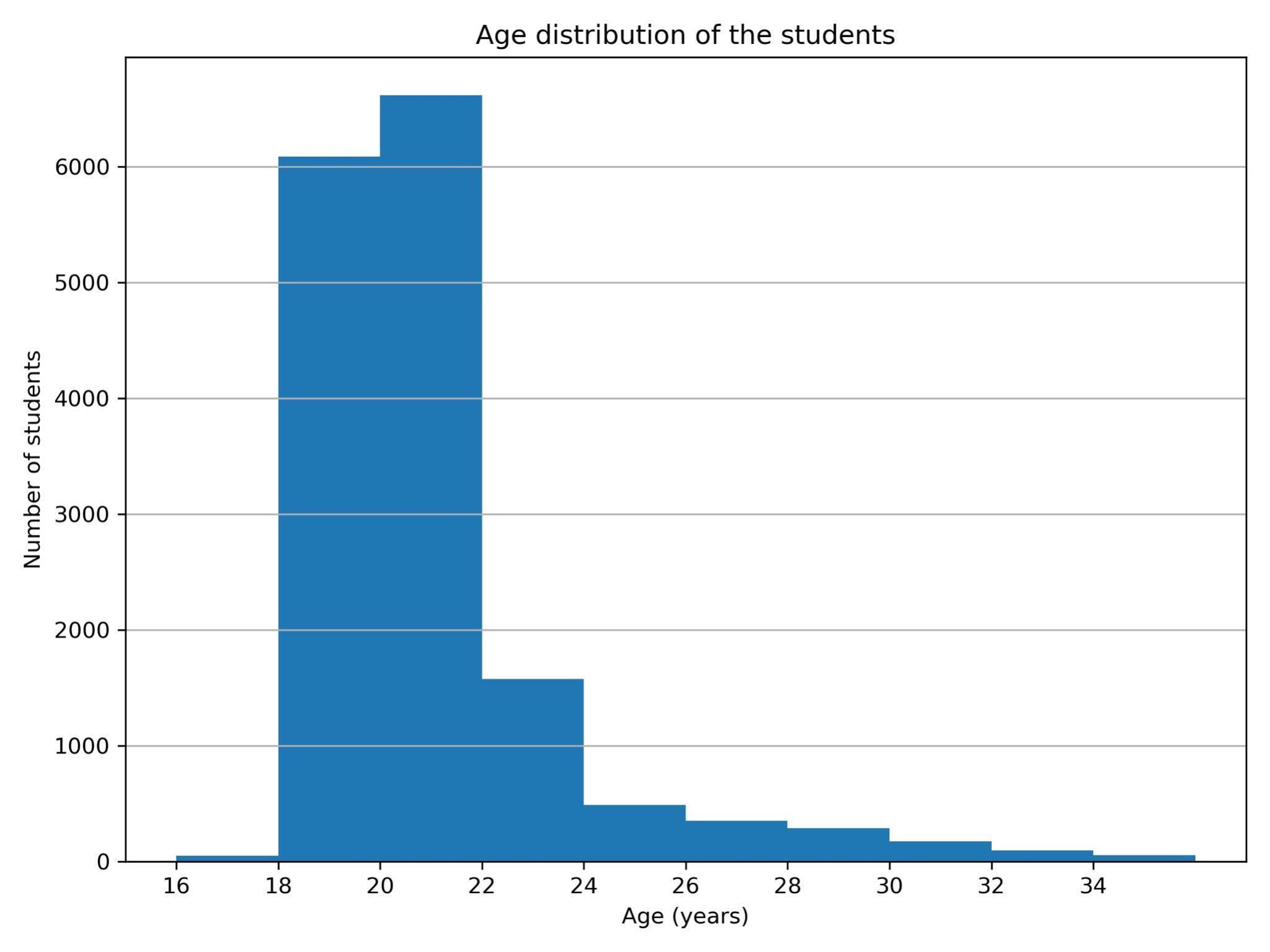
Fig C. Age distribution of students in the study population (n =15,800). Age was estimated from birth year. Mean age = 20.6 years; median age = 20 years; interquartile range = 2 years.

^
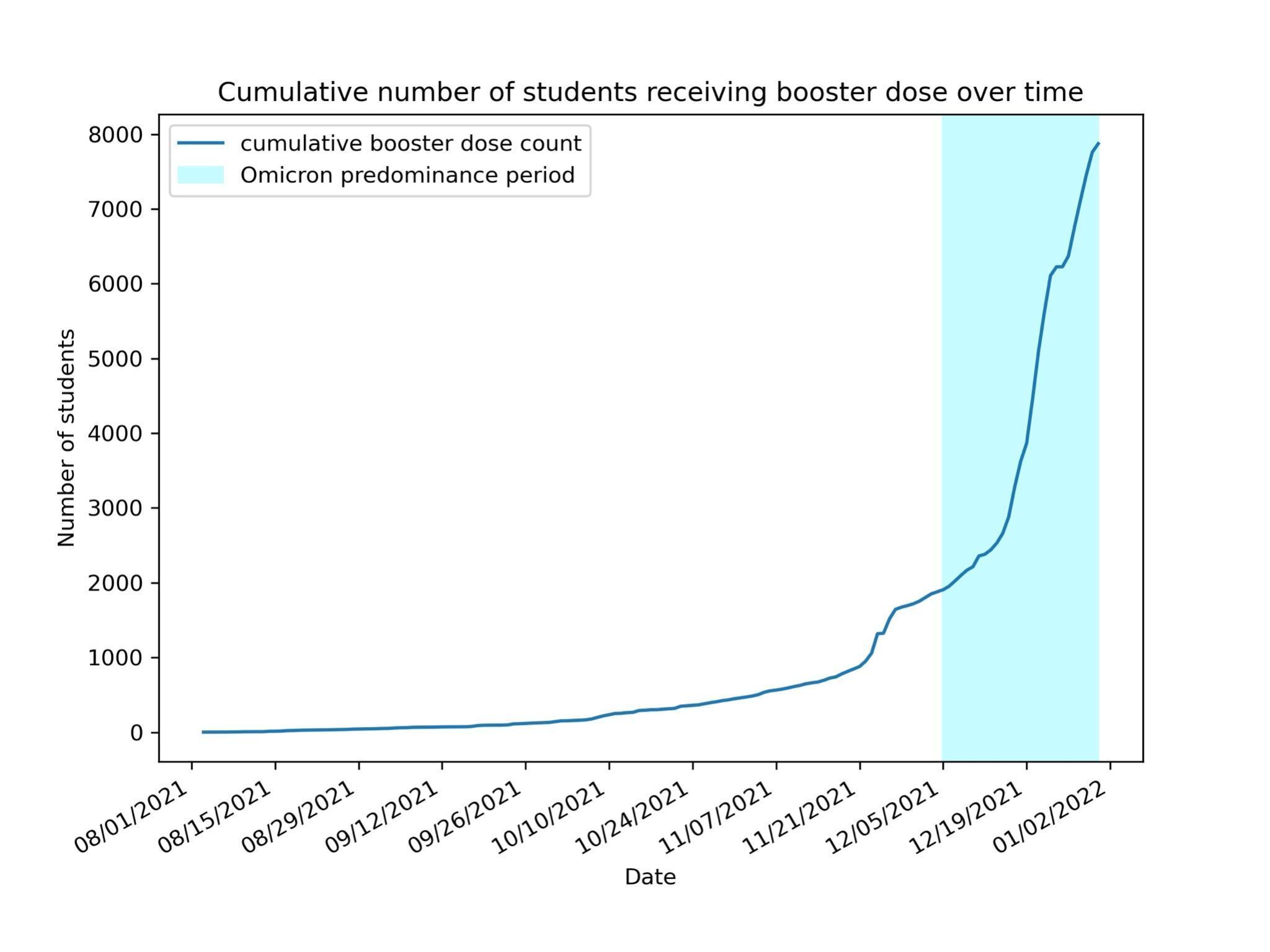
^*Fig D. Cumulative number of students receiving COVID-19 booster dose, over time. The shaded region represents the Omicron predominance period (December 5 to December 31, 2021).*
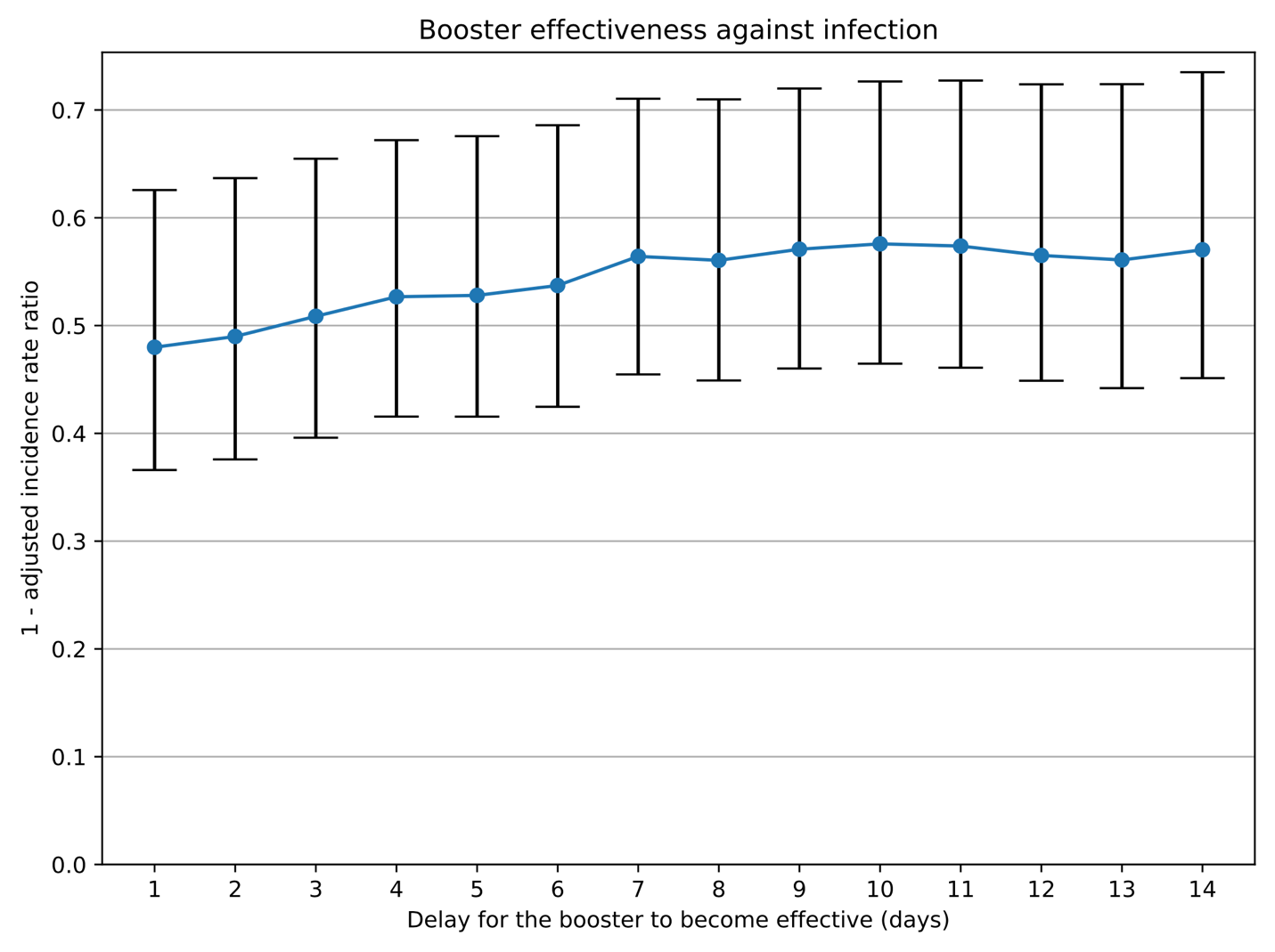
*Fig E. Mean and 95% confidence interval for the booster effectiveness against infection during the Omicron predominance period, as we vary the assumed delay for the booster dose to become effective after booster administration. Note that this analysis differs from our other sensitivity analysis considering the period shortly after booster administration: the analysis here combines all person days after the assumed delay into a single group, diluting the booster’s effect when the assumed delay is too short; the other sensitivity analysis separates people 0-6 days after booster administration from those that are 7+ days after.*

# **References for the Supplementary Appendix**

1. Cornell COVID-19 Modeling Team. Booster Effectiveness Against Omicron. 2022. Available from: https://covid.cornell.edu/_assets/files/booster-effectiveness-against-omicron-jan-27.pdf.

2. Frazier PI, Cashore JM, Duan N, Henderson SG, Janmohamed A, Liu B, et al. Modeling for COVID-19 college reopening decisions: Cornell, a case study. Proc Natl Acad Sci U S A. 2022;119(2):e2112532119. doi:10.1073/pnas.2112532119.

3. Cornell COVID-19 Modeling Team. Mathematical Modeling for Cornell’s Spring Semester. 2021. Available from: https://covid.cornell.edu/_assets/files/general-audience-spring-modeling-20210216.pdf.
